# Association of Renal Biomarkers with 3-Month and 1-Year Outcomes among Critically Ill Acute Stroke Patients

**DOI:** 10.1371/journal.pone.0072971

**Published:** 2013-09-13

**Authors:** Ying-Chih Huang, Yi-Ling Wu, Ming-Hsueh Lee, Jiann-Der Lee, Chih-Ying Wu, Huan-Lin Hsu, Ya-Hui Lin, Yen-Chu Huang, Wen-Hung Huang, Hsu-Huei Weng, Jen-Tsung Yang, Meng Lee, Bruce Ovbiagele

**Affiliations:** 1 Department of Neurology, Chang Gung University College of Medicine, Chang Gung Memorial Hospital, Chiayi, Taiwan; 2 Department of Neurosurgery, Chang Gung University College of Medicine, Chang Gung Memorial Hospital, Chiayi, Taiwan; 3 Department of Nephrology, Chang Gung University College of Medicine, Chang Gung Memorial Hospital, Linkou, Taiwan; 4 Department of Diagnostic Radiology, Chang Gung University College of Medicine, Chang Gung Memorial Hospital, Chiayi, Taiwan; 5 Department of Neurosciences, Medical University of South Carolina, Charleston, South Carolina, United States of America; Harvard Medical School, United States of America

## Abstract

**Background:**

The comparative relationships of widely recognized biomarkers of renal injury with short-term and long-term outcomes among critically ill acute stroke patients are unknown. We evaluated the impact of baseline albuminuria [urine albumin-creatinine ratio (UACR)≥30 mg/g] or low estimated glomerular filtration rate (eGFR<60 ml/min per 1.73 m^2^) on stroke patients admitted to the intensive care unit (ICU).

**Methods:**

We reviewed data on consecutive stroke patients admitted to a hospital ICU in Taiwan from September 2007 to August 2010 and followed-up for 1 year. Baseline UACR was categorized into <30 mg/g (normal), 30–299 mg/g (microalbuminuria), and ≥300 mg/g (macroalbuminuria), while eGFR was divided into ≥60, 45–59, and <45 ml/min per 1.73 m^2^. The outcome measure was death or disability at 3-month and 1-year after stroke onset, assessed by dichotomizing the modified Rankin Scale at 3–6 versus 0–2.

**Results:**

Of 184 consecutive patients, 153 (83%) met study entry criteria. Mean age was 67.9 years and median admission NIHSS score was 16. Among the renal biomarkers, only macroalbuminuria was associated with poorer 3-month outcome (OR 8.44, 95% CI 1.38 to 51.74, P = 0.021) and 1-year outcome (OR 18.06, 95% CI 2.59 to 125.94, P = 0.003) after adjustment of relevant covariates. When ischemic and hemorrhagic stroke were analyzed separately, macroalbuminuria was associated with poorer 1-year outcome among ischemic (OR 17.10, 95% CI 1.04 to 280.07, P = 0.047) and hemorrhagic stroke patients (OR 1951.57, 95% CI 1.07 to 3561662.85, P = 0.048), respectively, after adjustment of relevant covariates and hematoma volume.

**Conclusions:**

Presence of macroalbuminuria indicates poor 3-month and 1-year outcomes among critically ill acute stroke patients.

## Introduction

Albuminuria and low estimated glomerular filtration rate (eGFR), the two most widely recognized indices of kidney dysfunction, have separately been linked to poorer outcomes after an index stroke [1], [2], [3], [4],[5]. However, studies assessing the relationship of these renal biomarkers to acute stroke outcomes have generally been focused on albuminuria or eGFR, only one stroke type (hemorrhagic or ischemic), and short-term outcomes [5],[6],[7],[8]. Furthermore, there are very few data on the prognostic role of these measures of kidney injury among acute stroke patients who are critically ill requiring high level care within an intensive care unit [6]. We therefore conducted a hospital-based study to elucidate whether baseline biomarkers of kidney dysfunction are independently associated with short-term and long-term clinical outcomes among acute critically ill stroke patients.

## Methods

### Ethics Statement

This study was performed according to a protocol approved by the institutional review boards of Chang Gung Memorial Hospital, Chiayi, Taiwan. Written informed consent was obtained from patients who were able to understand our explanation. Patients with impaired consciousness or global aphasia were regarded as lack of capacity to consent by themselves. In this situation, a written informed consent was obtained from a first-degree relative (e.g. spouse or children). Local ethics committee approved this consent procedure.

### Patient Population

We prospectively collected data on consecutive patients with acute stroke within 72 hours from symptom onset, admitted to a Neurological and Neurosurgery Intensive Care Unit in a hospital in Taiwan from September 1, 2007 to August 31, 2010. Outcomes were assessed for all enrolled patients with modified Rankin Scale at 3 months and 1 year by a study nurse blinded to baseline kidney function. Both ischemic stroke and hemorrhagic stroke types were included. Patients with known impairment of functional status (mRS≥3) prior to the index stroke were excluded. Patients with subarachnoid hemorrhage and traumatic intracranial hemorrhage were also excluded. All acute stroke patients received brain computerized tomography and/or magnetic resonance imaging, and were evaluated by neurologists or neurosurgeons in the Emergency Department before admission to the Neurological and Neurosurgery Intensive Care Unit. Established hospital protocol criteria for admission to our Neurological and Neurosurgery Intensive Care Unit included hemodynamic instability, acute respiratory failure or intubation for airway protection, unstable neurologic status or Glasgow coma score (GCS)<11. We collected baseline demographic and clinical information for all patients, including sex, age, and cardiovascular risk factors such as hypertension, diabetes mellitus, atrial fibrillation, a history of stroke, systolic and diastolic blood pressure on admission. We also collected stroke characteristics including stroke type (ischemic or hemorrhagic), National Institutes of Health Stroke Scale (NIHSS) score on admission, and duration of intensive care unit and hospital stay.

Serum creatinine was obtained during the Emergency Department encounter. GFR was estimated by Chronic Kidney Disease Epidemiology Collaboration (CKD-EPI) equation adjusted for Asians [9]. Urine creatinine, and urine albumin levels were obtained on the second day of hospitalization based on random morning spot urine collection, and urine albumin-creatinine ratio (UACR) was calculated accordingly. Serum complete blood count, sodium, potassium, albumin, total cholesterol, LDL-cholesterol, admit blood glucose, HbA1C, and urine routine were also obtained. Hematoma volume for hemorrhagic stroke patients and ischemic stroke subtypes based on TOAST criteria for ischemic stroke patients were recorded. The surgical intervention, such as hematoma evacuation, or craniectomy, was also recorded.

The outcome measure was death or disability at 3-month and 1-year after stroke onset, assessed by dichotomizing the mRS at 3–6 versus 0–2.

### Statistical Analysis

We categorized eGFR into 3 groups according to the classification by the National Kidney Foundation, with slight modification: eGFR≥60, 45–59, and <45 ml/min per 1.73 m^2^ [10], [11], [12]. UACR was categorized into 3 groups: <30 mg/g (normal), 30–299 mg/g (microalbuminuria), and ≥300 mg/g (macroalbuminuria) [1], [12]. Presence of kidney dysfunction was defined as low eGFR (<60 ml/min per 1.73 m^2^) or increased UACR (≥30 mg/g) or both [13].

Clinical characteristics by presence of kidney dysfunction, UACR level, and eGFR level were compared using the chi-square test for categorical variables, and independent t-test or Mann-Whitney test for continuous or scoring variables, as appropriate. Multivariate-adjusted odds ratios (OR) and 95% confidence intervals (CI) for the study outcomes were calculated by a logistic regression analysis. The multivariate model included the following potential confounding factors: age, sex, admit NIHSS, admit systolic blood pressure, and surgical intervention. Hematoma volume was further adjusted when hemorrhagic stroke was analyzed separately. In the multivariate model for the urinary albumin level, the eGFR level was additionally adjusted. In the multivariate model for the eGFR level, the urinary albumin level was additionally adjusted. A P-value<0.05 was considered to be significant.

## Results

Among 184 consecutive stroke patients admitted to Neurology and Neurosurgery Intensive Care Unit during this period of time, 4 patients were excluded due to admission after 72 hours of stroke onset, 25 patients were excluded due to unknown mRS or mRS≥3 before index stroke, and 2 patients were excluded due to urine creatinine was missing. For the 153 critically ill acute stroke patients meeting study entry criteria, mean age was 67.9 (±13.3) years, median admission NIHSS score was 16 (interquartile range 9 to 21) and 131 (85.6%) had presence of kidney dysfunction on admission. Among 84 ischemic stroke patients, 26 (31.0%) were classified as large vessel atherosclerosis, 40 (47.6%) as cardioembolism, 2 (2.4%) as lacune, and 16 (19.0%) as undetermined. Among 69 hemorrhagic stroke patients, the median hematoma volume was 76.2 cc (interquartile range 28.6 to 172.0 cc). Thirteen (15.5%) ischemic stroke patients received craniectomy and 32 (46.4%) hemorrhagic stroke patients received hematoma evacuation.

Table 1 shows the baseline characteristics of the patients with and without presence of kidney dysfunction. Compared to those without kidney dysfunction, patients with presence of kidney dysfunction were older, had a higher frequency of hypertension, a higher admission systolic blood pressure, and a higher admit NIHSS score. Baseline characteristics according to the urinary albumin level and the eGFR level are shown in Table 2.

**Table 1:** The clinic characteristics of the patients with and without kidney dysfunction.

	Normal kidney function (n = 22)	Kidney dysfunction (n = 131)	P
Age,y, mean±SD	61.50±12.0	69.1±13.1	0.004
Female, n (%)	7(31.8)	50(38.2)	0.569
Comorbidity, n (%)			
Old Stroke	4(18.2)	38(29.0)	0.292
Hypertension	10(45.5)	99(75.6)	0.004
Diabetes	3(13.6)	33(25.2)	0.237
Atrial fibrillation	3(13.6)	28(22.1)	0.364
Stroke Type			
Ischemic	16(72.7)	68(51.9)	0.069
Hemorrhage	6(27.3)	63(48.1)	0.069
Blood Pressure, mmHg, mean±SD			
Systolic	164.1±30.3	181.9±37.5	0.037
Diastolic	91.1±20.7	100.3±23.0	0.077
eGFR, mL/min/1.73 m^2^,mmHg, median(IQR)	90.0(82.0–102.8)	67.6(47.6–84.5)	<0.001
UACR, mg/g, median(IQR)	11.1(7.3–23.1)	164.1(78.3–516.1)	<0.001
NIHSS on admission, median(IQR)	9.5(6.5–18)	16(10–21)	0.009
Baseline Lab, mean±SD			
Glucose, mg/dl	140.8±53.1	161.3±74.6	0.219
HbA1C, %	5.8%±0.9%	6.2%±1.4%	0.246
LDL-Cholesterol, mg/dl	112.3±46.3	113.8±33.9	0.860
WBC, *10^3^	7.8±2.4	9.1±3.2	0.076
Albumin, g/dl	3.8±0.6	3.6±0.6	0.182
Neurophil, *10^3^	5.1±2.6	6.4±3.0	0.061
ICU stay, day, median(IQR)	7(5–9.3)	8 (5–15.25)	0.144
Hospital stay, day, median(IQR)	24(13.8–54.5)	26(17–43.5)	0.634

Kidney dysfunction was defined as low eGFR (<60 ml/min per 1.73 m^2^) or increased UACR (≥30 mg/g) or both.

SD = standard deviation; mRS = modified Rankin Scale; eGFR = estimated glomerular filtration rate; IQR = interquartile range; UACR = urine albumin-creatinine ratio; NIHSS = The National Institutes of Health Stroke Scale; HbA1C = Glycated Hemoglobin; LDL = Low-density lipoprotein; WBC = white blood cell.

**Table 2:** The clinical characteristics of the patients according to the urinary protein level and eGFR level.

	Urinary protein level (mg/g)			
	<30	30–299	≥300	
	(n = 31)	(n = 73)	(n = 49)	*P*
Age, years, mean±SD	63.8±11.5	69.6±12.5	68.4±14.8	0.116
Female, n (%)	10 (32.3)	27 (37.0)	20 (40.8)	0.741
Comorbidity, n (%)				
Old stroke	6 (19.4)	22 (30.1)	14 (28.6)	0.518
Hypertension	16 (51.6)	55 (75.3)	38 (77.6)	0.025
Diabetes	5 (16.1)	14 (19.2)	17 (34.7)	0.078
Atrial fibrillation	5 (16.1)	18 (24.7)	9 (18.4)	0.538
Stroke Type				
Ischemic	23(74.2)	41(56.2)	20(40.8)	0.013
Hemorrhage	8(25.8)	32(43.8)	29(59.2)	0.013
Blood pressure on admission				
Systolic blood pressure, mmHg, mean±SD	164.8±28.0	176.5±37.8	192.7±36.8	0.003
Diastolic blood pressure, mmHg, mean±SD	91.1±19.4	97.9±22.3	105.7±24.2	0.016
eGFR, ml/min/1.73 m^2^, mmHg, median (IQR)	82.2(70.3–94.9)	72.7(55.9–90.6)	60.1(38.9–82.9)	<0.001
NIHSS on admission, median (IQR)	10 (6–18)	16 (10–21)	17 (13–23)	0.004

	eGFR (ml/min/1.73 m^2^)			
	≥60	45–59	≤45	
	(n = 75)	(n = 38)	(n = 40)	*P*
Age, years, mean±SD	64.5±12.9	71.5±10.0	71.4±14.8	0.004
Female, n (%)	30 (40.0)	16 (42.1)	11 (27.5)	0.324
Comorbidity, n (%)				
Old stroke	17 (22.7)	13 (34.2)	12 (30.0)	0.394
Hypertension	48 (64.0)	27 (71.1)	34 (85.0)	0.060
Diabetes	16 (21.3)	7 (18.4)	13 (32.5)	0.281
Atrial fibrillation	10 (13.3)	10 (26.3)	12 (30.0)	0.072
Stroke Type				
Ischemic	33(44.0)	27(71.1)	24(60.0)	0.018
Hemorrhage	42(56.0)	11(28.9)	16(40.0)	0.018
Blood pressure on admission				
Systolic blood pressure, mmHg, mean±SD	178.0±35.7	172.8±37.2	187.9±38.4	0.179
Diastolic blood pressure, mmHg mean±SD	97.5±21.9	93.8±18.7	106.8±26.4	0.031
Urinary protein level, mg/g, median (IQR)	78.7(24.7–262.8)	125.7(42.1–338.3)	306.1(118.4–3173.1)	<0.001
NIHSS at admission, median (IQR)	15 (7–21)	13.5 (7.8–18.3)	17.5 (15–25)	0.004

SD = standard deviation; mRS = modified Rankin Scale; eGFR = estimated glomerular filtration rate; IQR = interquartile range; UACR = urine albumin-creatinine ratio; NIHSS = The National Institutes of Health Stroke Scale; IQR, interquartile range.

Table 3 shows the impact of presence of kidney function and individual renal biomarker type/level on 3-month and 1-year outcomes among all acute critically ill stroke patients. Multivariate-adjusted logistic regression analysis showed that 3-month and 1-year outcomes were not significantly different between stroke patients with and without baseline presence of kidney dysfunction. Among the renal biomarkers, only macroalbuminuria was associated with poorer 3-month outcome (OR 8.44, 95% CI 1.38 to 51.74, P = 0.021) and 1-year outcome (OR 18.06, 95% CI 2.59 to 125.94, P = 0.003) after adjustment of relevant covariates. On the other hand, no significant relationship was observed between baseline eGFR level and 3-month and 1-year outcomes (Table 3). When ischemic and hemorrhagic stroke were analyzed separately, macroalbuminuria was associated with poorer 1-year outcome among ischemic (OR 17.10, 95% CI 1.04 to 280.07, P = 0.047) and hemorrhagic stroke patients (OR 1951.57, 95% CI 1.07 to 3561662.85, P = 0.048), respectively, after adjustment of relevant covariates and hematoma volume. When we further analyzed ischemic stroke subtypes based on TOAST criteria, there was no significant association between macroalbuminuria and 1-year outcome among large vessel stroke and cardioembolic stroke patients, respectively.

**Table 3:** The relationship between baseline subcategories of UACR or eGFR and poor outcome (modified Rankin Scale 3–6) after stroke.

	3-month poor outcome			1-year poor outcome		
*All stroke*						
	% of poor outcome	OR (95% CI)	P	% of poor outcome	OR (95% CI)	P
Kidney status						
Normal kidney function, N = 22	63.6	1.00(reference)		59.1	1.00(reference)	
Kidney dysfunction, N = 131	84.0	1.31(0.40–4.33)	0.658	83.2	1.52(0.45–5.08)	0.499
UACR, mg/g						
<30, N = 31	61.3	1.00(reference)		54.8	1.00(reference)	
30∼299, N = 73	79.5	1.06(0.34–3.35)	0.918	79.5	2.05(0.66–6.33)	0.214
≥300, N = 49	95.9	8.44(1.38–51.74)	0.021	95.9	18.06(2.59–125.94)	0.003
eGFR, ml/min per 1.73 m^2^						
≥60, N = 75	74.7	1.00(reference)		73.3	1.00(reference)	
45∼59, N = 38	78.9	1.33(0.43–4.09)	0.618	78.9	1.22(0.38–3.95)	0.734
<45, N = 40	95.0	1.96(0.36–10.83)	0.438	92.5	0.97(0.21–4.59)	0.969
***Ischemic stroke***						
Kidney Status						
Normal kidney function, N = 16	68.8	1.00(reference)		75.0	1.00(reference)	
Kidney dysfunction, N = 68	82.4	1.17(0.25–5.39)	0.841	82.4	0.70(0.13–3.67)	0.671
UACR, mg/g						
<30, N = 23	69.6	1.00(reference)		69.6	1.00(reference)	
30∼299, N = 41	78.0	0.62(0.12–3.12)	0.56	80.5	1.22(0.26–5.81)	0.800
≥300, N = 20	95.0	16.18(0.91–287.62)	0.058	95.0	17.10(1.04–280.07)	0.047
eGFR, ml/min per 1.73 m^2^						
≥60, N = 33	66.7	1.00(reference)		72.7	1.00(reference)	
45∼59, N = 27	81.5	3.23(0.62–16.85)	0.146	81.5	1.28(0.26–6.20)	0.763
<45, N = 24	95.8	5.48(0.46–65.26)	0.178	91.7	1.14(0.14–8.99)	0.905
***Hemorrhagic stroke***						
Kidney Status						
Normal kidney function, N = 6	50.0	1.00(reference)		16.7	1.00(reference)	
Kidney dysfunction, N = 63	85.7	6.11(0.46–80.90)	0.170	84.1	113.46(1.58–8140.51)	0.030
UACR, mg/g						
<30, N = 8	37.5	1.00(reference)		12.5	1.00(reference)	
30∼299, N = 32	81.3	4.14(0.31–55.15)	0.282	78.1	225.83 (0.27–186654.63)	0.114
> = 300, N = 29	96.6	19.76(0.67–585.53)	0.084	96.6	1951.57 (1.07–3561662.85)	0.048
eGFR, ml/min per 1.73 m^2^						
> = 60, N = 42	81.0	1.00(reference)		73.8	1.00(reference)	
45∼59, N = 11	72.7	0.62(0.04–10.05)	0.739	72.7	0.98 (0.04–25.84)	0.992
<45, N = 16	93.8	2.77(0.09–81.96)	0.556	93.8	17.49 (0.23–1318.78)	0.195

Kidney dysfunction was defined as low eGFR (<60 ml/min per 1.73 m^2^) or increased UACR (≥30 mg/g) or both. UACR = urine albumin-creatinine ratio; eGFR = estimated glomerular filtration rate.

* Multivariate adjusted for sex, age, NIHSS at admission, baseline SBP, and surgical intervention. In the multivariate model for the urinary albumin level, the eGFR level was additionally adjusted. In the multivariate model for the eGFR level, the urinary albumin level was additionally adjusted. Hematoma volume was further adjusted when hemorrhagic stroke was analyzed separately.

## Discussion

We found that presence of macroalbuminuria at baseline among acute critically stroke patients admitted to a Neurological and Neurosurgery Intensive Care Unit was independently associated with poor outcomes at three month and one year. Indeed, the relation of macroalbuminuria and 3-month and 1-year poor prognosis in these patients was independent of renal filtration function (eGFR), and we did not find a significant relationship between any level of eGFR vs. outcome after adjusting for albuminuria. These latter observations support the notion that these two renal biomarkers (albuminuria and renal filtration function) likely make or are associated with distinct pathophysiologic contributions to stroke outcomes. Published studies suggest that proteinuria independently contributes to the increased risks of neurologic deterioration, mortality, and short-term poor functional outcome, but the eGFR may not be relevant to these outcomes after ischemic stroke [7], [8]. Moreover, with regard to incident stroke risk, one study showed that UACR was the kidney biomarker most strongly associated with risk of incident stroke [14], while indirect comparisons of results from meta-analyses also imply that the strength of the association of albumiuria with stroke risk is greater than that seen with low eGFR [15], [16], [17]. Current stratification of chronic kidney disease stage is based on the levels of GFR levels and people with albuminuria only are classified into early stage of chronic kidney disease [11]. Taken together, measure of glomerular permeability is at least as importance as glomerular filtration when we evaluate kidney function among stroke patients as well as general population.

In this study, baseline microalbuminuria (UACR between 30 to 299 mg/g) was not an independent predictor for poor 3-month or 1-year outcomes. It is not clear whether UACR≥30 mg/g is necessarily the optimal cutoff point for poor prognosis in acute critically ill stroke patients. While one study of hemorrhagic stroke patients in the intensive care unit suggested that UACR>200 mg/g was independently associated with unfavorable neurologic outcome at discharge [6], another study revealed UACR≥100 mg/g to be an independent predictor of higher hospital mortality and longer hospital stay in critically ill patients with general medical conditions [18]. To evaluate an optimal cutoff point of UACR for poor 3-month and 1-year outcomes among acute critically ill stroke patients was beyond the scope of this study but the cutoff point of UACR to link to poor outcome among critically ill stroke patients is likely to be higher than in the general population based on the current and relevant studies.

The effect size of baseline macroalbuminuria was larger on 1-year outcome than 3-month outcome among critically ill acute stroke patients. Short term outcomes (e.g. 3 months) tend to more directly reflect the circumstances leading up to or surrounding the index stroke, while long term outcomes (one year and beyond) tend to reflect chronic ongoing underlying problems (e.g. widespread endothelial dysfunction, multiple comorbidities, risk factor control, etc) as well as the effects of the index stroke. Presence of macroalbuminuria as a prognosticator probably reflects a variety of underlying factors that are deleterious to optimal recovery after a stroke. It may be a ready reflection of generalized systemic vasculopathy [19], [20], [21], [22], a marker of widespread endothelial dysfunction [23], [24], or an indicator of profound inflammation and oxidative stress [25], [26]. Previous studies have shown that macroalbuminuria is a powerful and independent predictor of poor prognosis in various conditions including heart failure [27], diabetes mellitus [28], [29], and general population [30]. The current study affirmed macroalbuminuria to be a prognosticator among critically ill acute stroke patients.

There are several limitations in this study. First, we have no information about the pre-morbid kidney status of these patients, and both UACR and eGFR may have been influenced by severity of the index stroke. In fact, both increased UACR and low eGFR were associated higher baseline NIHSS scores at baseline, but still after adjustment of relevant covariates including NIHSS score only macroalbuminuria independently predicted poorer 3-month and 1-year outcomes. Second, we only evaluated UACR and eGFR once after stroke. The day-to-day variability of UACR within individuals is high and a single sample may not accurately characterize the true level of albumin excretion. Third, the sample size of study was modest and all patients were enrolled from a single hospital. Fourth, the study population was acute critically stroke patients admitted to the Intensive Care Unit and the results may not necessarily be generalizable to other patient groups. The study is strengthened by its simultaneous assessment both indices of kidney dysfunction (UACR and eGFR) within the same stroke patient population, evaluation by investigators experienced in stroke severity and functional status assessment, and inclusion of a long term endpoint.

In conclusion, baseline macroalbuminuria is associated with poor 3-month and 1-year outcomes among critically ill acute stroke patients, even after adjusting for several confounders including renal filtration function and stroke severity. Baseline low eGFR does not appear to be a prognosticator in these patients. It is unknown whether the relationship between albuminuria and poor stroke outcomes is definitively an epiphenomenon or in some way causal. Since modulators of the renin-angiotensin system mitigate progression of albuminuria [31], [32], [33], [34], it may be worthwhile to evaluate the impact of these agents on enhancing outcomes among critically-ill acute stroke patients with macroalbuminuria, in a randomized controlled trial.
